# Seroprevalence of *Leptospira* in Racehorses and Broodmares in New Zealand

**DOI:** 10.3390/ani10111952

**Published:** 2020-10-23

**Authors:** Charlotte F. Bolwell, Chris W. Rogers, Jackie Benschop, Julie M. Collins-Emerson, Brooke Adams, Katherine R. Scarfe, Erica K. Gee

**Affiliations:** 1 School of Veterinary Science, Massey University, Palmerston North 4442, New Zealand; C.W.Rogers@massey.ac.nz (C.W.R.); J.Benschop@massey.ac.nz (J.B.); J.M.Collins-Emerson@massey.ac.nz (J.M.C.-E.); B.Adams@massey.ac.nz (B.A.); E.K.Gee@massey.ac.nz (E.K.G.); 2 School of Agriculture and Environment, Massey University, Palmerston North 4442, New Zealand; 3 IDEXX Laboratories (NZ) ULC, School of Veterinary Science Complex, Massey University, Palmerston North 4442, New Zealand; Katherine-Scarfe@idexx.com

**Keywords:** horse, leptospirosis, risk factors, epidemiology

## Abstract

**Simple Summary:**

Leptospirosis is a zoonotic disease, caused by bacteria (*Leptospira*), that is frequently reported to occur in horses worldwide. Leptospirosis has been associated with abortions in mares, can cause a painful eye condition, and it poses a zoonotic risk to equine workers and veterinarians. Epidemiological data on the occurrence of leptospirosis or the frequency of exposure to the bacteria in horses in New Zealand are lacking. A survey was conducted to determine the seroprevalence of *Leptospira* in Thoroughbred racing and breeding horses in New Zealand. Horse owners were surveyed, and a blood sample was taken from the horses enrolled in the study to determine the frequency of five different types of *Leptospira* found to cause leptospirosis in humans and livestock in New Zealand. The results showed that a quarter of the horses sampled had previously been exposed to *Leptospira*. Several management factors, such as grazing horses alternately with cattle or sheep, increasing horse age, and breeding horses, were linked to exposure to *Leptospira* in this group of horses. Given the level of exposure found, horses may play a role in the epidemiology of leptospirosis in New Zealand.

**Abstract:**

A cross-sectional survey was conducted to determine the seroprevalence of *Leptospira* in a cohort of horses and to evaluate potential risk factors for *Leptospira* seropositivity in horses in New Zealand. The convenience sample included 499 Thoroughbred racing and breeding horses from 25 commercial properties in North Island, New Zealand. A questionnaire was used to collect demographic data on horses and property-level information on grazing and management practices, pest (rodent) management, access to natural waterways, other livestock on the property, and possible contact with wildlife. The microscopic agglutination test was used to test sera for serovars Ballum, Copenhageni, Hardjo (bovis), Pomona, and Tarassovi. Logistic regression was used to investigate the risk factors for *Leptospira* seropositivity to at least one serovar and for each serovar individually. A total of 124 (25%, 95% confidence interval (CI) 21–29%) horses had positive titres to any one of the five serovars. The seroprevalence of Ballum, Copenhageni, Hardjo (bovis), Pomona, and Tarassovi was 5% (95% CI 3–7%), 9% (95% CI 7–12%), 6% (95% CI 4–8%), 6% (95% CI 4–8%), and 6% (95% CI 4–8%), respectively. Broodmares, compared to racehorses and alternately grazing horses with sheep, increased the odds of exposure to any one serovar, whilst grazing the same time as sheep and alternately grazing horses with cattle increased the odds of exposure to Ballum and Hardjo (bovis), respectively. Historical exposure to *Leptospira* in racing and breeding horses was identified, and risk factors were consistent with pasture-based exposure.

## 1. Introduction

Leptospirosis is a zoonotic disease that is of increasing concern within the public health in New Zealand [1]. The incidence of human cases notified in New Zealand over the last five years (2014–2018) increased from 1.2 cases per 100,000 in 2014 to 2.3 cases per 100,000 in 2018, peaking at 2.9 cases per 100,000 in 2017 [2]. The serovars reported in human cases are consistent with those reported in livestock and rodents in New Zealand, and there is a strong occupational association (meat workers and farmers) in human cases [3]. *Leptospira* are endemic in livestock in New Zealand, with seroprevalence ranging from 3% to 90% depending on serovar and species studied [4,5].

As seen in other species, *Leptospira* infection in horses can cause fever, depression, generalised pain [6,7], and subclinical infection can occur [6,8]. Additionally, *Leptospira* infection in horses has been associated with the development of equine recurrent uveitis (ERU), which can occur months to years after the initial infection [8,9]. Infection in pregnant mares has been reported to result in pregnancy loss, with abortions and stillborn foals occurring late in gestation [6,10].

The prevalence of *Leptospira* in horses varies worldwide (from 1–95%) depending on the serovar and the geographical location studied [6,11]. Data on the seroprevalence of *Leptospira* in horses in New Zealand are scant. One study reported the frequency of different types of serovars ranging from 1% to 15%, from 762 horse samples submitted to three animal health laboratories during 1989 and 1990 [12]. Surveillance reports from 1974–1990 indicated that leptospirosis was confirmed in four mares with abortions [13], and another report indicated that infection with *Leptospira interrogans* serovar Pomona was associated with two cases of mare abortions in 1998 [14]. More recently, surveillance reports indicated a likely infection with *Leptospira* in two cases of ERU [15,16]. However, there are no recent scientific studies with a focus on *Leptospira* and horses in New Zealand.

The Thoroughbred breeding and racing industries are the major equestrian industries in New Zealand, with over 5000 mares bred and 3000 races run on average per season [17,18]. Due to the temperate climate of New Zealand, breeding horses are kept and grown at pasture all year round [17,19]. The common pasture management practices of broodmares (breeding mares) on commercial stud farms involves alternately grazing them with other livestock, including sheep and cattle [20]. Therefore, as horses in New Zealand are not vaccinated against *Leptospira*, the pasture-based management of horses provides an opportunity for exposure to the *Leptospira* serovars found in other grazing livestock [21] and wildlife species (such as possums, rats, rabbits, mustelids, or hedgehogs) in New Zealand [22].

Given the increasing incidence of human notifications, the apparent change in the epidemiology of leptospirosis in New Zealand [23], and the lack of recent data on the prevalence of *Leptospira* in horses in New Zealand, a study was initiated to provide current data on the prevalent serovars within horses in New Zealand. The objectives of the study were to determine the seroprevalence of *Leptospira* in a cohort of horses and collect horse- and property-level information to evaluate potential risk factors for *Leptospira* seropositivity in horses in New Zealand.

## 2. Materials and Methods 

### 2.1. Study Design

A cross-sectional study was conducted to investigate the prevalence of *Leptospira* serovars in Thoroughbred racehorses and broodmares and to assess the risk factors associated with *Leptospira* seropositivity. The sampling frame consisted of Thoroughbred commercial stud farms and racing stables located in the Manawatu and commercial stud farms located in the Waikato and Auckland regions of North Island, New Zealand; there is a regional concentration of Thoroughbred racing and breeding horses in these locations [24]. Contact details for trainers and stud masters were obtained from the New Zealand Thoroughbred Racing and the Thoroughbred Breeders’ Association, respectively. Trainers and stud masters were contacted by telephone to provide them with information about the project and to invite them to participate in the study. The inclusion criteria for sampling were (1) the horse was a broodmare or a racehorse in active work or training and (2) owner’s consent for enrolment of their horses in the study. For each trainer or stud master that agreed to take part, a convenience sample of horses was selected from each property dependent on meeting the inclusion criteria. A sample size calculation for a prevalence survey (with an assumed design effect of 2) indicated that 277 samples would provide 80% power and 95% confidence to detect a seroprevalence of 10% [12].

### 2.2. Sample Collection and Serological Testing

The blood sampling of horses as part of this study was approved by the Massey University Animal Ethics Committee, Massey University, Palmerston North (Protocol number 16/36). Visits to each equine property and sampling of horses took place from June–September 2016, and a written consent form was received from all trainers and stud masters prior to sampling. Blood samples (2 × 10 mL red top vacutainer and 20 g vacutainer needle; (Becton Dickenson Limited (BD), Auckland, New Zealand) were collected once from the jugular vein of each horse into 10-mL vacuum tubes. Samples were placed in a cooled and insulated transport container or refrigerated at 4–8 °C until submitted to a commercial veterinary diagnostic laboratory (IDEXX Laboratories Ltd., Massey University, Palmerston North) for serum extraction and testing. All samples were submitted within four days of sampling.

A microscopic agglutination test (MAT) as described by Faine [25] was used to initially detect antibodies against *Leptospira interrogans* serovar Pomona, and any remaining serum was stored at −80 °C and later thawed in a refrigerator for testing of the remaining endemic strains in New Zealand: *Leptospira interrogans* serovar Copenhageni and *Leptospira borgpetersenii* serovars Ballum, Hardjo (bovis), and Tarassovi (no other serovars were tested for, as they were considered exotic to New Zealand [22]). Briefly, serum samples were diluted 1/6.25 in phosphate-buffered saline (PBS; Lorne-buffered saline tablets 0.9% NaCl, Lorne Laboratories Ltd., Reading, UK), and doubling dilutions were made to obtain a final series ranging from 1/25 to 1/3200, inclusive of the addition of the antigen. Antigen strains were purchased from the Institute of Environmental Science and Research (ESR), Porirua, New Zealand. Standard antisera were from the World Organisation for Animal Health (OIE), Reference Laboratory for Leptospirosis (Amsterdam, The Netherlands). The end point titre was recorded at the highest dilution where at least 50% agglutination occurred.

### 2.3. Questionnaire

A face-to-face questionnaire was designed to capture information from trainers and stud masters about exposure to potential horse- and property-level risk factors; the questionnaire was completed by one of the authors (B.A.) at the time of sampling the horses. The questionnaire covered demographic information such as sex, age, role (racehorse or broodmare) of the horse, vaccination status against other pathogens, and whether the horse had been previously diagnosed with leptospirosis (Supplementary Table S1). Property-level information included region; type of property (stud farm or training stables); approximate size of property; general horse management systems; storage of feed; pest (rodent) management; any previous history of leptospirosis in livestock; horses’ access to natural waterways; contact with other livestock on the property; evidence of wildlife on the property (including trainers sighting rats, mice, possums, hedgehogs, rabbits, and mustelids (ferrets/stoats/weasels) on the property); and whether other animals (cattle, sheep, deer, or dogs) on the property were vaccinated against *Leptospira* (Supplementary Table S2). The surveying of respondents in this study was evaluated by peer review and judged to be low risk; approval by the Massey University Human Ethics Committee was not required.

### 2.4. Statistical Analysis

The breeding records for all the broodmares included in the study were obtained from the New Zealand Thoroughbred Racing online database to determine if the mare had a history of reproductive losses such as a miss (mare not detected pregnant at day 45 after covering) or slip (mare is diagnosed as pregnant and, subsequently, found to be empty, or mare is observed to abort a foetus) within the seven years prior to the 2016/17 breeding season. These data were summarised, and a prevalence ratio was calculated comparing horses with and without a history of reproductive losses. All data were entered into a Microsoft Excel spreadsheet and checked for errors and outliers. Horse age was summarised as the median and interquartile range (IQR), whilst categorical data were summarised as count and percentages. A frequency graph was generated to visualise the distribution of antibody titres (lowest to highest) by serovar type. Seroprevalence and 95% confidence intervals (CI) were calculated using a titre cut-off ≥50. The MAT titre cut-off (≥50) was chosen as recommended by Blackmore et al. [26], which, in the New Zealand setting, gives high sensitivity and reasonable specificity indicative of exposure. Horse and property-level variables were summarised as the count and percentage of horses positive to at least one serovar tested and to each serovar individually.

All analyses were conducted in Stata version 14 (StataCorp LP, College Station, TX, USA). Logistic regression was used to investigate possible horse and property-level risk factors for *Leptospira* seropositivity to at least one serovar and for each serovar individually. Variables showing association with the outcome (*p* ≤ 0.2) in a univariable analysis were analysed in multivariable regression models fitted using backwards elimination. Variables were retained in multivariable models based on a likelihood ratio *p*-value of *p* ≤ 0.05 or if there was evidence of a confounding variable that altered the odds ratios in the final model by more than 20% [27]. To adjust for the potential clustering of horses within a property, models were run using the variance-covariance matrix VCE (cluster) option in Stata to allow for intergroup correlation at the property level when investigating positivity to at least one serovar (no clustering at the property-level was present for the other outcomes investigated). Possible correlations between the property-level variables were investigated, and when two variables were considered to be measuring the same factor and were found to be associated with each other, only one of the variables was used in the multivariable model. Biologically plausible interactions were tested between variables significant in the final models. The final models were assessed using the Hosmer-Lemeshow chi-squared goodness-of-fit test.

## 3. Results

### 3.1. Population Description

Samples were taken from 500 horses, of which the sample from one horse was subsequently excluded, as it did not meet the inclusion criteria. In total, 499 horses were sampled across 25 properties (Table 1), of which 80% (400/499) of the horses were female, and 67% (335/499) of the population sampled were broodmares. Overall, the median age of the horses tested was eight years (IQR 5–12 years), whilst the median age of racehorses and broodmares was four (IQR three–six) and 10 (IQR 8–14) years, respectively. A few horses were reported to have had a previous eye condition (7/480), but none were reported to have had recurrent uveitis. Of the broodmares with a previous breeding record (86%; 287/335), 13% (37/287) previously had an abortion, and 45% (129/287) had a miss or slip during the last seven years.

### 3.2. Serovar Results

**Figure 1:**
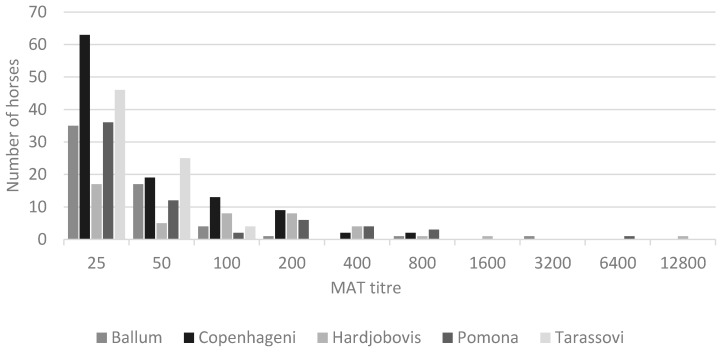
Frequency of microscopic agglutination titres (cut-off of ≥25) for Ballum, Copenhageni, Hardjo (bovis), Pomona, or Tarassovi in a convenience sample of 499 racehorses and broodmares in New Zealand.

The seroprevalence of Pomona, Hardjo (bovis), Ballum, Copenhageni, and Tarassovi was 13% (95% CI 10–16%), 9% (95% CI 7–12%), 12% (95% CI 9–15%), 22% (95% CI 18–26%), and 15% (95% CI 12–18%), respectively, at any titre level. The frequency of titres for each of the serovars is shown in Figure 1, with the highest titres being recorded for Copenhageni and Hardjo (bovis).

**Figure 2:**
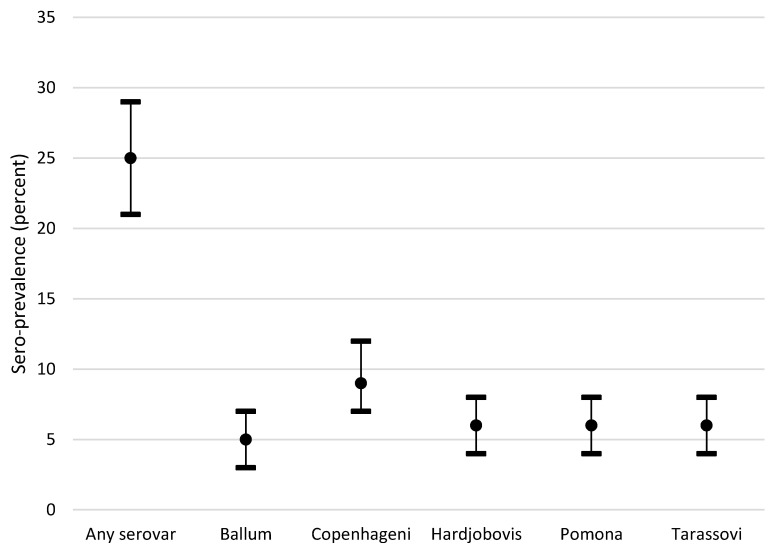
Point estimates and 95% confidence intervals for the prevalence of microscopic agglutination titres ≥50 to Ballum, Copenhageni, Hardjo (bovis), Pomona, Tarassovi, or to any one of the serovars tested in a convenience sample of 499 racehorses and broodmares in New Zealand.

The prevalence of each serovar using a positive cut-off MAT titre of ≥50 is shown in Figure 2. A total of 124/499 (25%, 95% CI 21–29%) horses had positive titres at ≥50 to any one of the five serovars, with 24 horses positive to >1 serovar.

Overall, at least one horse was positive to any one of the serovars on 24/25 properties (Table 1).

### 3.3. Questionnaire Results

None of the horses or any of the other livestock were previously diagnosed with leptospirosis. On one property, it was reported that an owner previously tested seropositive to *Leptospira*, and on a further property, the respondent reported “my husband had leptospirosis”; no further details were provided about these potential cases. The median age of horses positive to any serovar was 11 (IQR 7–14) years compared to seven (IQR 5–11) years for horses that were negative. Just under half (45.9%; 17/37) of the broodmares that previously had an abortion were positive to at least one of the serovars tested, with 32% (80/250) of horses that did not have an abortion being positive to at least one serovar (prevalence ratio 1/4). The number and percentage of horses stratified by the horse- and property-level variables collected in the questionnaire is shown in Table 1 for any serovar and for each serovar individually.

### 3.4. Logistic Regression Analysis

#### 3.4.1. Any Serovar

The results of the univariable regression for seropositivity to any of the serovars tested are shown in Table 2 (correlated variables not shown).

The variables significantly associated with seropositivity to any serovar in the multivariable analysis are shown in Table 3.

In the final model, the adjusted odds of seropositivity increased with the increasing horse age, and the adjusted odds were higher for broodmares compared to racehorses and if horses were grazed alternately with sheep (Table 3). Flooding on the property in the last 12 months reduced the adjusted odds of seropositivity to any one of the serovars tested. The inclusion of the evidence of hedgehogs on the property, whilst not significant in the final model, adjusted the odds ratios of the other variables and improved the overall model fit. The Hosmer-Lemeshow goodness of fit test value for the final model was *p* = 0.29, indicating no evidence of poor fit of data to the model.

#### 3.4.2. Serovar Ballum

The role of the horse, the presence of natural water on a property, and horses grazing at the same time as sheep were significantly associated with increased odds of seropositivity to Ballum in the univariable analysis (Supplementary Table S3). In the final model, the adjusted odds of seropositivity to Ballum were three times greater for broodmares compared to racehorses and for horses grazing at the same time as sheep (Table 3).

#### 3.4.3. Serovar Copenhageni

Increasing horse age, broodmares, and horse contact with deer over a fence were significantly associated with increased odds of seropositivity to Copenhageni; evidence of mustelids on the property was associated with reduced odds in the univariable analysis (Supplementary Table S4). In the final model, increasing horse age was associated with increasing adjusted odds of seropositivity, and evidence of mustelids on the property was associated with lower adjusted odds (Table 3). The Hosmer-Lemeshow *p*-value for the final model was *p* = 0.25.

#### 3.4.4. Serovar Hardjo (Bovis)

The following variables were associated with increased odds of seropositivity in the univariable analysis: age; the location horse feed was stored prior to opening; the presence of goats on the property; and horses grazing with cattle at the same time, grazing alternately with cattle, and grazing alternately with sheep (Supplementary Table S5). Flooding on the property in the last 12 months was associated with reduced odds of seropositivity to Hardjo (bovis). There was a significant association with horses grazing alternately with cattle in the final model (OR 10.2, 95% CI 1.3–77.6). (Table 3). No significant association was shown for horses grazing alternately with sheep, but this variable adjusted the odds ratio for grazing alternately with cattle and was, therefore, retained in the final model.

#### 3.4.5. Serovar Pomona

In the univariable analysis, there was a significant association between the seropositivity of Pomona and increasing horse age, role of the horse, flooding in the last 12 months, the presence of goats, horses grazing with cattle at the same time, and horses grazing alternately with cattle (Supplementary Table S6). After adjusting for the other variables in the multivariable model, only the increasing age was significantly associated with seropositivity to Pomona (OR 1.19, 95% CI 1.10–1.29; *p* < 0.001).

#### 3.4.6. Serovar Tarassovi

Role, age of the horse, and horses grazing alternately with cattle were significantly associated with increased odds of seropositivity of Tarassovi in the univariable analysis, whilst evidence of rabbits on the property was associated with reduced odds (Supplementary Table S7). After adjusting for the other variables in the multivariable model, only increasing age was associated with seropositivity to Tarassovi (OR 1.15, 95% CI 1.06–1.24; *p* < 0.001).

## 4. Discussion

This is the first study to investigate the seroprevalence of *Leptospira* in racing and breeding horses in New Zealand. As horses are not vaccinated for *Leptospira* in New Zealand, our results showed widespread natural exposure to the five serovars tested in this cohort of apparently healthy horses. There was a higher prevalence in broodmares compared to racehorses, and several risk factors for exposure to *Leptospira* were identified.

A quarter of the horses in the cohort tested positive to at least one serovar, which is the same as the seroprevalence rates reported in Korea (25%) [28] and slightly lower than a study in Australia (29%) [29]. Studies in Brazil, Italy, and North America have reported higher rates of 45%, 67%, and 77% of horses testing positive to at least one serovar, respectively [30,31,32]. Direct comparisons of seroprevalence across geographic regions may be difficult due to variations in the positive titre cut-off used between studies and differences in endemic serovars across regions.

In agreement with serological surveys of horses in Europe and North America [31,33,34], the horses in this study showed positivity to more than one serovar. Copenhageni was the most common serovar reported in this study (9%), with a similar prevalence (6%) reported for Hardjo (bovis), Pomona, and Tarassovi. A previous serological survey of routine blood samples from horses submitted to animal health laboratories in New Zealand in 1988 and 1989–1990 [12] found Hardjo (bovis), Copenhageni, and Bratislava to be the most prevalent serovars; Tarassovi was not tested, and Bratislava was assumed by the authors to be a cross-reaction to Pomona (a recognised paradoxical cross-reaction), as Bratislava is not endemic in New Zealand. Pomona has previously been reported in cases of sporadic abortions [13,14] and, more recently, in a case of renal disease and two cases of ERU [15,16]. Pomona is reported to be the most common serovar causing clinical disease, such as ERU and abortions, in North America [34,35]. The most common serovars in horses in North America, Brazil, and Italy are Australis and Bratislava [30,31,32,36]. Neither of these serovars are considered endemic in New Zealand [3].

This study identified broodmares as having a higher risk of exposure to any serovar, and to Copenhageni and Ballum, compared to racehorses. Consistent with reports from other countries [33,34,36,37], age was also identified as being associated with an increased risk of exposure for several of the serovar-specific multivariable models. The temperate climate of New Zealand allows broodmares to be managed, and youngstock to be raised, year-round at pasture (in paddocks/fields) [38]. The management of broodmares on stud farms is related to their age and stage of production, with empty (not pregnant) mares kept in paddocks of relatively low pasture cover during the wetter seasons compared to pregnant mares and those with foals at foot [20]. Additionally, intensive stocking rates for nonpregnant mares results in increased grazing pressures and mares grazing closer to the ground and in latrine areas [20,39]. The combination of these management factors provides a potential mechanism for broodmare exposure to *Leptospira* in the soil. In contrast, once in training, racehorses typically have less than 12 h access to pasture per day and experience a more “intensive” management system [24,40]. Our findings are in agreement with those from Switzerland [33] and Brazil [30], reporting an increased risk of exposure to *Leptospira* for horses spending more time at pasture and for horses in extensive rearing systems.

The pasture-based management of broodmares on stud farms frequently involves co- or cross-grazing with other livestock [19]. In this study, alternating grazing horses with sheep or cattle and grazing horses at the same time as sheep were associated with a higher exposure to any serovar, Hardjo (bovis), and Ballum, respectively. Hardjo (bovis) and Pomona are prevalent serovars in cattle and sheep in New Zealand [3], and species-specific shedding rates of 21% for cattle and 32% for sheep have been reported [41]. Furthermore, seropositivity to Copenhageni, Ballum, and Tarassovi has recently been reported for sheep, beef cattle, and deer in New Zealand [21]. In agreement with the results of this study, co-grazing with cattle or other livestock also increased the risk of exposure to *Leptospira* for horses in South Africa [42]. Contact with livestock in neighbouring properties over fences or via shared drinking water sources was reported as a risk factor for horses in Brazil [36], though these specific factors were not significantly associated with the risk of exposure in the current study.

The prevalence of the serovars tested was lower than the estimated 25% used for the sample size calculations for this study, which may have impacted on the power to detect some serovar-specific risk factors. Despite this, many of the risk factors identified in this study were consistent with those reported for other cohorts of horses worldwide. It should be noted that horses are not located on a property for life, and there is seasonal movement of both racehorse and broodmares between properties [43]. The breed registration requirement for the natural service of the mares does mean that mares are transported and managed on the breeding farms where the stallions are based during breeding, but all mares return to the original stud farm soon after mating [17]. Most of the broodmares sampled are managed on these properties for a number of seasons, thus providing successive exposure to the property-level risk factors.

Flooding and increased rainfall have also been identified as risk factors for exposure to *Leptospira* in horses [32,44]. In contrast, the results of this study showed a reduced risk of exposure to any serovar with flooding. It is speculated that there may be other management factors associated with the management of broodmares on stud farms, such as moving horses away or fencing off flooded areas to reduce pugging [45], that were not measured in this study, which may have contributed to the lower odds observed. However, further work to determine farm-specific risk factors for exposure to *Leptospira* is required.

As found in the current study, similar studies in Kansas [32] and Switzerland [33] reported high titres in the horses sampled that could be consistent with active infection, but no clinical signs of disease were reported in the horses. High antibody levels do not indicate if a horse is currently shedding *Leptospira*, as horses can be both seropositive or negative and be shedding the bacteria [8,32]. Although no inference about active infections in the cohort of horses reported in this study can be made, horses with active infections that are shedding may be a risk to both animals and people in contact with them [35]. Two respondents in this study reported a family member with a history of leptospirosis, but further details on the diagnosis and infecting serovars of the human cases were beyond the scope of the current study. A study conducted in Kentucky reported some seroconversion in equine veterinarians and equine farm workers [46]. High-risk occupations for leptospirosis in New Zealand include farmers, cattle workers, and abattoirs, but, to date, there have been no specific investigations of the rates of disease in equine-related occupations in New Zealand.

## 5. Conclusions

The study identified historical exposure to *Leptospira* in racing and breeding horses in New Zealand and provided baseline data that can be used for future studies on *Leptospira* in horses in New Zealand. Risk factors for exposure to *Leptospira* were identified that were consistent with the pasture-based management of horses in New Zealand. Given these findings, horses may be a potential risk to other animals, particularly livestock that they may be co- or cross-grazed with, and to those working in the racing and breeding industries in New Zealand. Further work is needed to identify if horses in New Zealand actively shed *Leptospira* and the extent of exposure in the wider population of horses in New Zealand.

## Figures and Tables

**Table 1:** The number and percentage of horses positive to any *Leptospira* serovar (at cut-off ≥50), and each serovar individually stratified by horse- and property-level variables obtained from a convenience sample of racehorse trainers and stud masters.

Variable	Level	Any Serovar		Ballum	Copenhageni	Hardjo (Bovis)	Pomona	Tarassovi
		Total Horses	Number of Horses Positive (%)	Number of Horses Positive (%)	Number of Horses Positive (%)	Number of Horses Positive (%)	Number of Horses Positive (%)	Number of Horses Positive (%)
Horse-level								
Sex	Female	400	111 (27.8)	22 (5.5)	41 (10.3)	22 (5.5)	27 (6.8)	28 (7.0)
	Male	99	13 (13.1)	2 (2.0)	4 (4.0)	6 (6.1)	1 (1.0)	1 (1.0)
Role	Broodmare	335	103 (30.8)	21 (6.3)	38 (11.3)	20 (6.0)	26 (7.8)	27 (8.1)
	Racehorse	164	21 (12.8)	3 (1.8)	7 (4.3)	8 (4.9)	2 (1.2)	2 (1.2)
Vaccinated against other pathogens *	No/unsure	105	14 (13.3)	1 (0.9)	6 (5.7)	6 (5.7)	0	2 (1.9)
	Yes	394	110 (27.9)	23 (5.8)	39 (9.9)	22 (5.5)	28 (7)	27 (6.8)
Property-level								
Region	Manawatu	315	72 (22.8)	14 (4.4)	25 (5.9)	19 (6.0)	17 (5.4)	16 (5.1)
	Waikato	136	40 (29.4)	7 (5.2)	16 (11.8)	7 (5.2)	8 (5.9)	10 (7.4)
	Auckland	48	12 (25)	3 (6.3)	4 (8.3)	2 (4.2)	3 (6.3)	3 (6.3)
Natural water source on property	No	69	17 (24.6)	1 (1.5)	6 (8.7)	5 (7.3)	5 (7.3)	4 (5.8)
	Yes	430	107 (24.9)	23 (5.4)	39 (9.1)	23 (5.4)	23 (5.4)	25 (5.8)
Natural water source type *	Bore	2	2 (100)	1 (50.0)	1 (50.0)	0 (0.0)	2 (100)	1 (50.0)
	River	211	55 (26.1)	11 (5.2)	18 (8.5)	12 (5.7)	12 (5.7)	11 (5.2)
	Stream	16	4 (25)	1 (6.3)	3 (18.8)	0 (0.0)	0 (0.0)	1 (6.3)
	Creek	193	42 (21.8)	9 (4.7)	15 (7.8)	11 (5.7)	9 (4.7)	10 (5.2)
	Swamp	8	4 (50)	1 (12.5)	2 (25.0)	0 (0.0)	0 (0.0)	2 (25.0)
Flooding on property in the last 12 months	No	259	73 (28.2)	12 (4.6)	27 (10.4)	19 (7.3)	18 (7.0)	16 (6.2)
	Yes	240	51 (21.2)	12 (5.0)	18 (7.5)	9 (3.8)	10 (4.2)	13 (5.4)
Evidence of wildlife on the property								
Rats	No	86	16 (18.6)	3 (3.5)	5 (5.8)	5 (5.8)	5 (5.8)	4 (4.7)
	Yes	413	108 (26.2)	21 (5.1)	40 (9.7)	23 (5.6)	23 (5.6)	25 (6.1)
Mice	No	9	9 (11.1)	0 (0.0)	1 (11.1)	0 (0.0)	0 (0.0)	0 (0.0)
	Yes	490	124 (24.9)	24 (4.9)	44 (9.0)	28 (5.7)	28 (5.7)	29 (5.9)
Possums	No	114	24 (21.1)	6 (5.3)	9 (7.9)	4 (3.5)	6 (5.3)	5 (4.4)
	Yes	385	100 (26.0)	18 (4.7)	36 (9.4)	24 (6.2)	22 (5.7)	24 (6.2)
Hedgehogs	No	246	53 (21.5)	13 (5.3)	21 (8.5)	12 (4.9)	12 (4.9)	11 (4.5)
	Yes	253	71 (28.1)	11 (4.4)	24 (9.5)	16 (6.3)	16(6.3)	18 (7.1)
Rabbits	No	19	6 (31.6)	1 (5.3)	0 (0.0)	2 (10.5)	0 (0.0)	3 (15.8)
	Yes	480	118 (24.6)	23 (4.8)	45 (9.4)	26 (5.4)	28 (5.8)	26 (5.4)
Mustelids	No	364	92 (25.3)	21 (5.8)	38 (10.4)	18 (5.0)	20 (5.5)	21 (5.8)
	Yes	135	32 (23.7)	3 (2.2)	7 (5.2)	10 (7.4)	8 (5.9)	8 (5.9)
Traps for rodents on property	No	19	2 (10.5)	0 (0.0)	0 (0.0)	2 (10.5)	0 (0.0)	0 (0.0)
	Yes	480	122 (25.4)	24 (5.0)	45 (9.4)	26 (5.4)	28 (5.8)	29 (6.0)
Feed storage prior to opening	Feed shed	333	85 (25.5)	17 (5.1)	33 (9.9)	15 (4.5)	21 (6.3)	20 (6.0)
	Other shed	50	11 (22.0)	2 (4.0)	3 (6.0)	3 (6.0)	4 (8.0)	3 (6.0)
	Shed and silo	44	8 (18.2)	1 (2.3)	2 (4.6)	6 (13.6)	0 (0.0)	1 (2.3)
	Other (silo/kegs)	72	20 (27.8)	4 (5.6)	7 (9.7)	4 (5.6)	3 (4.2)	5 (6.9)
Feed storage once opened	Open feed bags	72	17(23.6)	7 (9.7)	9 (12.5)	0 (0.0)	5 (6.9)	3 (4.2)
	Unsealed feed bins	73	15 (20.6)	2 (2.7)	5 (6.9)	6 (8.2)	2 (2.7)	3 (4.1)
	Sealed feed bins	230	53 (23.0)	10 (4.4)	17 (7.4)	12 (5.2)	11 (4.8)	14 (6.1)
	Silo	85	26 (30.6)	3 (3.5)	7 (8.2)	8 (9.4)	8 (9.4)	6 (7.1)
	Other	39	13 (33.3)	2 (5.1)	7 (18.0)	2 (5.1)	2 (5.1)	3 (7.7)
Animals on property								
Cats	No	64	13 (20.3)	3 (4.7)	4 (6.3)	2 (3.1)	5 (7.8)	3 (4.7)
	Yes	435	111 (25.5)	21 (4.8)	41 (9.4)	26 (6.0)	23 (5.3)	26 (6.0)
Dogs	No	22	5 (22.7)	1 (4.6)	3 (13.6)	0 (0.0)	0 (0.0)	2 (9.1)
	Yes	477	119 (25.0)	23 (4.8)	42 (8.8)	28 (5.9)	28 (5.9)	27 (5.7)
Pigs	No	499	124 (24.9)	24 (4.8)	45 (9.0)	28 (5.6)	28 (5.6)	29 (5.8)
	Yes	0	-	-	-	-	-	-
Goats	No	465	112 (24.1)	24 (5.2)	42 (9.0)	24 (5.2)	23 (5.0)	27 (5.8)
	Yes	34	12 (35.3)	0 (0.0)	3 (8.8)	4 (11.8)	5 (14.7)	2 (5.9)
Dairy cattle	No	383	95 (24.8)	19 (5.0)	36 (9.4)	19 (5.0)	21 (5.5)	21 (5.5)
	Yes	116	29 (25.0)	5 (4.3)	9 (7.8)	9 (7.8)	7 (6.1)	8 (6.9)
Beef cattle	No	101	14 (13.9)	3 (3.0)	7 (6.9)	4 (4.0)	1 (1.0)	2 (2.0)
	Yes	398	110 (27.6)	21 (5.2)	38 (9.5)	24 (6.0)	27 (6.8)	27 (6.8)
Sheep	No	217	46 (21.2)	5 (2.3)	19 (8.8)	9 (4.2)	10 (4.6)	11 (5.1)
	Yes	282	78 (27.6)	19 (6.7)	26 (9.2)	19 (6.7)	18 (6.4)	18 (6.4)
Deer	No	451	110 (24.4)	22 (4.9)	37 (8.2)	26 (5.8)	26 (5.8)	26 (5.8)
	Yes	48	14 (29.2)	2 (4.2)	8 (16.7)	2 (4.2)	2 (4.2)	3 (6.3)
Animals on property vaccinated for lepto ^	No	305	73 (23.9)	14 (4.6)	31 (10.1)	12 (3.9)	16 (5.3)	19 (6.2)
	Yes	130	31 (23.9)	9 (6.9)	12 (9.2)	7 (5.4)	7 (5.4)	5 (3.9)
	Do not know	64	20 (31.3)	1 (1.6)	2 (3.1)	9 (14.1)	5 (7.8)	5 (7.8)
Contact with other animals								
Contact with cattle	No	77	9 (11.7)	2 (2.6)	5 (6.5)	1 (1.3)	1 (1.3)	1 (1.3)
	Yes	422	115 (27.3)	22 (5.2)	40 (9.5)	27 (6.4)	27 (6.4)	28 (6.6)
Graze horses same time as cattle	No	321	76 (23.7)	14 (4.4)	28 (8.8)	14 (4.4)	14 (4.4)	19 (5.9)
	Yes	178	48 (27.0)	10 (5.6)	17 (9.6)	14 (7.9)	14 (7.9)	10 (5.6)
Graze horses alternately with cattle	No	151	26 (17.2)	8 (5.3)	16 (10.6)	1 (0.7)	5 (3.3)	4 (2.7)
	Yes	348	98 (28.2)	16 (4.6)	29 (8.3)	27 (7.8)	23 (6.6)	25 (7.2)
Horses share water source with cattle	No	277	63 (22.7)	16 (5.8)	25 (9.0)	10 (3.6)	14 (5.1)	13 (4.7)
	Yes	222	61 (27.5)	8 (3.6)	20 (9.0)	18 (8.1)	14 (6.3)	16 (7.2)
Contact with cattle over fence	No	300	69 (23.0)	17 (5.7)	27 (9.0)	12 (4.8)	17 (5.7)	14 (4.7)
	Yes	199	55 (27.6)	7 (3.5)	18 (9.1)	16 (8.0)	11 (5.5)	15 (7.5)
Contact with sheep	No	68	6 (8.8)	0 (0.0)	4 (5.9)	1 (1.5)	0 (0.0)	1 (1.5)
	Yes	431	118 (27.4)	24 (5.6)	41 (9.5)	27 (6.3)	28 (6.5)	28 (6.5)
Graze horses same time as sheep	No	340	86 (25.3)	13 (3.8)	30 (8.8)	18 (5.3)	18 (5.3)	22 (6.5)
	Yes	159	38 (23.9)	11 (6.9)	15 (9.4)	10 (6.3)	10(6.3)	7 (4.4)
Graze horses alternately with sheep	No	271	61 (22.5)	11 (4.1)	28 (10.3)	9 (3.3)	14 (5.2)	14 (5.2)
	Yes	228	63 (27.6)	13 (5.7)	17 (7.5)	19 (8.3)	14 (6.1)	15 (6.6)
Horses share water source with sheep	No	365	90 (24.7)	17 (4.7)	36 (9.9)	16 (4.4)	20 (5.5)	21 (5.8)
	Yes	134	34 (25.4)	7 (5.2)	9 (6.7)	12 (9.0)	8 (6.0)	8 (6.0)
Contact with sheep over fence	No	371	94 (25.3)	18 (4.9)	38 (10.2)	18 (4.9)	23 (6.2)	20 (5.4)
	Yes	128	30 (23.4)	6 (4.7)	7 (5.5)	10 (7.8)	5 (3.9)	9 (7.0)
Contact with deer	No	269	58 (21.6)	14 (5.2)	22 (8.2)	13 (4.8)	12 (4.5)	14 (5.2)
	Yes	230	66 (28.7)	10 (4.3)	23 (10.0)	15 (6.5)	16 (7.0)	15 (6.5)
Graze horses same time as deer	No	499	124 (24.9)	24 (4.8)	45 (9.0)	28 (5.6)	28 (5.6)	29 (5.8)
	Yes	0	-	-	-	-	-	-
Graze horses alternately with deer	No	490	123 (25.1)	24 (4.9)	44 (9.0)	28 (5.7)	28 (5.7)	29 (5.9)
	Yes	9	1 (11.1)	0 (0.0)	1 (11.1)	0 (0.0)	0 (0.0)	0 (0.0)
Horses share water source with deer	No	490	123 (25.1)	24 (4.9)	44 (9.0)	28 (5.7)	28 (5.7)	29 (5.9)
	Yes	9	1 (11.1)	0 (0.0)	1 (11.1)	0 (0.0)	0 (0.0)	0 (0.0)
Contact with deer over fence	No	451	110 (24.4)	22 (4.9)	37 (8.2)	26 (5.8)	26 (5.8)	26 (5.8)
	Yes	48	14 (29.2)	2 (4.2)	8 (16.7)	2 (4.2)	2 (4.2)	3 (6.3)
Property	1	16	4 (25.0)	1 (6.3)	3 (18.8)	0 (0.0)	0 (0.0)	1 (6.3)
	2	12	2 (16.7)	0 (0.0)	0 (0.0)	2 (16.7)	0 (0.0)	0 (0.0)
	3	13	1 (7.7)	0 (0.0)	0 (0.0)	1 (7.7)	0 (0.0)	0 (0.0)
	4	7	0 (0.0)	0 (0.0)	0 (0.0)	0 (0.0)	0 (0.0)	0 (0.0)
	5	2	2 (100)	1 (50.0)	1 (50.0)	0 (0.0)	2 (100)	1 (50.0)
	6	21	7 (33.3)	1 (4.8)	1 (4.8)	2 (9.5)	2 (9.5)	2 (9.5)
	7	8	4 (50)	1 (12.5)	2 (25.0)	0 (0.0)	0 (0.0)	2 (25.0)
	8	14	1 (7.1)	0 (0.0)	1 (7.1)	0 (0.0)	0 (0.0)	0 (0.0)
	9	9	1 (11.1)	0 (0.0)	1 (11.1)	0 (0.0)	0 (0.0)	0 (0.0)
	10	18	2 (11.1)	1 (5.6)	0 (0.0)	0 (0.0)	1 (5.6)	0 (0.0)
	11	46	11 (23.9)	5 (10.9)	7 (15.2)	0 (0.0)	4 (8.7)	1 (2.2)
	12	12	3 (25)	1 (8.3)	2 (16.7)	1 (8.3)	0 (0.0)	1 (8.3)
	13	19	6 (31.6)	1 (5.3)	0 (0.0)	2 (10.5)	0 (0.0)	3 (15.8)
	14	11	4 (36.4)	0 (0.0)	2 (18.2)	2 (18.2)	2 (18.2)	1 (9.1)
	15	20	3 (15)	0 (0.0)	0 (0.0)	3 (15.0)	0 (0.0)	0 (0.0)
	16	12	1 (8.3)	0 (0.0)	0 (0.0)	0 (0.0)	0 (0.0)	1 (8.3)
	17	9	1 (11.1)	0 (0.0)	1 (11.1)	0 (0.0)	0 (0.0)	0 (0.0)
	18	21	6 (28.6)	2 (9.5)	0 (0.0)	2 (9.5)	1 (4.8)	1 (4.8)
	19	15	1 (6.7)	0 (0.0)	1 (6.7)	0 (0.0)	0 (0.0)	0 (0.0)
	20	5	1 (20)	0 (0.0)	1 (20.0)	0 (0.0)	0 (0.0)	0 (0.0)
	21	25	11 (44)	0 (0.0)	2 (8.0)	4 (16.0)	5 (20.0)	2 (8.0)
	22	67	23 (34.3)	5 (7.5)	7 (10.5)	5 (7.5)	6 (9.0)	5 (7.5)
	23	30	4 (13.3)	0 (0.0)	2 (6.7)	0 (0.0)	0 (0.0)	2 (6.7)
	24	39	13 (33.3)	2 (5.1)	7 (18.0)	2 (5.1)	2 (5.1)	3 (7.7)
	25	48	12 (25.0)	3 (6.3)	4 (8.3)	2 (4.2)	3 (6.3)	3 (6.3)

* Horses vaccinated for at least one of the following: tetanus, strangles, equine herpes virus, salmonella, or rotavirus. ^ were other animals on the property vaccinated against *Leptospira*. Horses are not vaccinated against *Leptospira* in New Zealand.

**Table 2:** Results of the univariable logistic regression investigating horse and property-level risk factors for positive microscopic agglutination tests (cut-off titre ≥50) to any of the *Leptospira* serovars tested in a convenience sample of racehorses and broodmares in New Zealand.

Risk Factor	Level	Odds Ratio	Robust SE	95% Confidence Interval	*p*-Value	Likelihood Ratio Test *p*-Value
Horse age		1.15	0.03	1.09–1.20	<0.001	<0.001
Role	Racehorse	Ref	-	-	-	<0.001
	Broodmare	3.02	0.79	1.80–5.05	<0.001	
Vaccinated for other pathogens	No/unsure	Ref	-	-	-	0.001
	Yes	2.52	0.77	1.37–4.61	0.003	
Region	Manawatu	Ref	-	-	-	0.34
	Waikato	1.41	0.33	0.89–2.21	0.14	
	Auckland	1.13	0.40	0.56–2.28	0.74	
Natural water source on property	No	Ref	-	-	-	0.97
	Yes	1.01	0.30	0.56–1.83	0.97	
Flooding on property in the last 12 months	No	Ref	-	-	-	0.07
	Yes	0.69	0.14	0.46–1.04	0.07	
Evidence of wildlife on the property						
Rats	No	Ref	-	-	-	0.13
	Yes	1.55	0.46	0.86–2.78	0.14	
Mice	No	Ref	-	-	-	0.30
	Yes	2.68	2.86	0.33–21.65	0.36	
Possums	No	Ref	-	-	-	0.27
	Yes	1.32	0.34	0.79–2.18	0.29	
Hedgehogs	No	Ref	-	-	-	0.09
	Yes	1.42	0.30	0.94–2.14	0.09	
Rabbits	No	Ref	-	-	-	0.50
	Yes	0.71	0.36	0.26–1.90	0.49	
Mustelids	No	Ref	-	-	-	0.72
	Yes	0.92	0.22	0.58–1.46	0.72	
Traps for rodents on property	No	Ref	-	-	-	0.11
	Yes	2.90	2.19	0.66–12.72	0.16	
Feed storage prior to opening	Feed shed	Ref	-	-	-	0.63
	Shed and silo	0.65	0.27	0.29–1.45	0.29	
	Other shed	0.82	0.30	0.40–1.68	0.59	
	Other	1.12	0.33	0.63–1.99	0.69	
Feed storage once opened	Sealed feed bins	Ref	-	-	-	0.40
	Open feed bags	1.03	0.33	0.55–1.93	0.92	
	Unsealed feed bins	0.86	0.28	0.45–1.65	0.66	
	Silo	1.47	0.42	0.85–2.56	0.17	
	Other	1.67	0.62	0.80–3.48	0.17	
Animals on property						
Cats	No	Ref	-	-	-	0.36
	Yes	1.34	0.44	0.70–2.56	0.37	
Dogs	No	Ref	-	-	-	0.81
	Yes	1.13	0.59	0.41–3.13	0.81	
Goats	No	Ref	-	-	-	0.16
	Yes	1.72	0.64	0.82–3.58	0.15	
Contact with other animals						
Graze horses same time as cattle	No	Ref	-	-	-	0.42
	Yes	1.19	0.25	0.78–1.81	0.42	
Graze horses alternately with cattle	No	Ref	-	-	-	0.008
	Yes	1.88	0.46	1.16–3.05	0.01	
Graze horses same time as sheep	No	Ref	-	-	-	0.74
	Yes	0.93	0.21	0.60–1.44	0.74	
Graze horses alternately with sheep	No	Ref	-	-	-	0.19
	Yes	1.31	0.27	0.88–1.97	0.19	
	Yes	0.90	0.22	0.56–1.44	0.67	
Contact with deer over fence	No	Ref	-	-	-	0.47
	Yes	1.28	0.43	0.66–2.47	0.47	

**Table 3:** Results of the final multivariable logistic regression models investigating horse and property-level risk factors for positive microscopic agglutination tests to any of the *Leptospira* serovars and to serovars Ballum, Copenhageni, and Hardjo (bovis).

Risk Factor	Level	Odds Ratio	Robust SE	95% Confidence Interval	*p*-Value
Any serovar					
Horse age		1.13	0.03	1.08–1.18	<0.001
Role	Racehorse	Ref	-	-	-
	Broodmare	1.73	0.46	1.03–2.93	0.04
Flooding on property last 12 months	No	Ref	-	-	-
	Yes	0.59	0.09	0.43–0.80	0.001
Graze horses alternately with sheep	No	Ref	-	-	-
	Yes	1.4	0.19	1.07–1.84	0.01
Evidence of hedgehogs on the property	No	Ref	-	-	-
	Yes	0.77	0.12	0.55–1.05	0.1
Ballum					
Role	Racehorse	Ref	-	-	-
	Broodmare	4.49	2.36	1.29–15.6	0.02
Grazing same time as sheep	No	Ref	-	-	-
	Yes	2.45	1.06	1.05–5.72	0.03
Copenhageni					
Horse age		1.14	0.04	1.07–1.22	<0.001
Ferrets on the property	No	-	-	-	-
	Yes	0.39	0.17	0.16–0.91	0.03
Hardjo (bovis)					
Horses graze alternately with cattle	No	-	-	-	-
	Yes	10.2	10.5	1.3–77.6	0.02
Horses graze alternately with sheep	No	-	-	-	-
	Yes	1.86	0.78	0.81–4.24	0.14

## Supplementary Materials

The following are available online at https://www.mdpi.com/2076-2615/10/11/1952/s1: Table S1: Horse questionnaire, Table S2: Farm questionnaire, Table S3: Results of univariable logistic regression investigating horse and property-level risk factors for seropositivity to Ballum in a convenience sample of 499 racehorses and broodmares in New Zealand, Table S4: Results of univariable logistic regression investigating horse and property-level risk factors for seropositivity to Copenhageni in a convenience sample of 499 racehorses and broodmares in New Zealand, Table S5: Results of univariable logistic regression investigating horse and property-level risk factors for seropositivity to Hardjo (bovis) in a convenience sample of 499 racehorses and broodmares in New Zealand, Table S6: Results of univariable logistic regression investigating horse and property-level risk factors for seropositivity to Pomona in a convenience sample of 499 racehorses and broodmares in New Zealand, Table S7: Results of univariable logistic regression investigating horse and property-level risk factors for seropositivity to Tarassovi in a convenience sample of 499 racehorses and broodmares in New Zealand.
