# Supplementary material for: Seroprevalence of Leptospira in Racehorses and Broodmares in New Zealand
Source: Animals (Basel). 2020 Oct 23;10(11):1952. doi: 10.3390/ani10111952 (PMC7690811; doi:10.3390/ani10111952)
Supplement: Supplementary file 1 [file animals-10-01952-s001.pdf]

animals

MDPI

Article

Seroprevalence of *Leptospira* in Racehorses and Broodmares in New Zealand

Charlotte F. Bolwell [1,*], Chris W. Rogers [1,2], Jackie Benschop [1], Julie M. Collins-Emerson [1], Brooke Adams [1], Katherine R. Scarfe [3] and Erica K. Gee [1]

[1] School of Veterinary Science, Massey University, Palmerston North, 4442, New Zealand; C.W.Rogers@massey.ac.nz (C.W.R.); J.Benschop@massey.ac.nz (J.B.); J.M.Collins-Emerson@massey.ac.nz (J.M.C.-E.); B.Adams@massey.ac.nz (B.A.); E.K.Gee@massey.ac.nz (E.K.G.)
[2] School of Agriculture and Environment, Massey University, Palmerston North, 4442, New Zealand
[3] IDEXX Laboratories (NZ) ULC, School of Veterinary Science Complex, Massey University, Palmerston North, 4442, New Zealand; Katherine-Scarfe@idexx.com (K.R.S.)
* Correspondence: c.bolwell@massey.ac.nz; Tel.: +64-6-356-9099

Received: 4 September 2020; Accepted: 20 October 2020; Published: date

Table S1. Horse questionnaire.

Participant's consent

I have read the Information Sheet and the details of the study have been explained to me. I have been informed about the study protocol including the questionnaire and use of the blood samples taken from horses.

I agree with the study protocol and consent to blood samples being taken from the horses I enroll in the study. I agree to answer the horse and farm questionnaire.

Signature………………………………………………….. Date ………………………………………………….

**Farm / Stable ID:**	**Date:**

**Property Details**			
Name			
Region			
Main property type	Stud farm		Training stable
Number of horses on property (including foals) (total)			
**Racing:** number of horses in work			
**Stud:** number of mares on property			
Size of property (h)			
	No	Yes	Don't know

Is the property close to natural water sources? (creek / river)	If yes, what?				
In the last 12 months, has there been any flooding on the property?	No		Yes		Don't know
Have you noticed any evidence of the following on the farm? How managed?	Rats		Mice		Possums
	Hedgehogs		Rabbits		Ferrets/stoats
Stud Farm: classes of livestock fed hard feed	Empty (Y/ N)	In foal broodmares (Y/ N)	Weanlings / yearlings (Y/ N) / (Y/ N)		
If yes, how is hard feed stored? Prior to use (Bags) (timeframe-weeks)	Feed shed	Other Shed	Other		
	In open feed bags in barn	In unsealed feed bins in barn	In sealed feed bins in barn		
In use	Other: please state				
Any of the following animals on the farm	Cat	Dog	Pigs	Goats	
	Dairy Cattle	Beef Cattle	Sheep	Deer	
	Rats	Mice	Other?		
If there are other animals, are they vaccinated for leptospirosis?	No		Yes		Don't know
	If yes, which?				
If there are other animals, have any of them been diagnosed with leptospirosis?	No		Yes		Don't know
	If yes, which?				
What type of contact do horses have with the following animals? (Tick)	None	Graze paddock same time	Alternately grazed	Share water source	Over fence
Cattle					
Sheep					
Deer					
Are you willing to be contacted again about this survey?	No		Yes		

Do you have any further comments?	

Table S2. Farm questionnaire.

Date:

Farm / stable ID: | **Horse ID:** | **Sample ID:**

Horse Details				
Name				
Age				
Sire				
Dam				
Brand				
Breed	TB		SB	
Sex	Female	Male	Gelding	
Horse role	Broodmare		Racehorse	
How long has this horse been on the property? (please state)				
**History**				
Diagnosis of leptospirosis in the past?	No		Yes	
	Comments:			
If mare: Been in foal before?	Yes / No<br>Find breeding record			
Currently in foal?	Yes / No			
History of foaling slips /abortions?	No		Yes	
	Comments:			
Any history of eye abnormalities/disease?	No	Uveitis diagnosed	Apparent blindness	Cloudiness/ opacity
	Other:			

Vaccination history:	Tetanus	Strangles	Herpes	Salmonella	Rotavirus
			Other:		

Table S3: Results of univariable logistic regression investigating horse and property-level risk factors for seropositivity to Ballum (at cut-off ≥50), in a convenience sample of 499 racehorses and broodmares in New Zealand.

**Risk Factor**	**Level**	**Odds Ratio**	**Robust SE**	**95% Confidence Interval**	***p*-Value**	**LR *p*-Value**
Age		1.05	0.04	0.97–1.14	0.25	0.26
Role	Racehorse	Ref	-		-	0.02
	Broodmare	0.28	0.17	0.08–0.95	0.04	
Vaccinated against other pathogens	No/unsure	Ref	-	-	-	0.02
	Yes	6.44	6.62	0.86–48.3	0.07	
Region	Manawatu	Ref	-		-	0.85
	Waikato	1.17	0.55	0.46–2.95	0.75	
	Auckland	1.43	0.94	0.40–5.18	0.58	
Natural water source on property	No	Ref	-		-	0.11
	Yes	3.84	3.96	0.51–28.92	0.19	
Flooding in the last 12 months	No	Ref	-		-	0.85
	Yes	1.08	0.45	0.48–2.46	0.85	
Evidence of wildlife on the property						
Rats	No	Ref	-		-	0.51
	Yes	1.48	0.93	0.43–5.08	0.53	
Possums	No	Ref	-		-	0.80
	Yes	0.88	0.43	0.34–2.28	0.80	
Hedgehogs	No	Ref	-		-	0.62
	Yes	0.81	0.34	0.36–1.86	0.63	
Rabbits	No	Ref	-		-	0.93
	Yes	0.91	0.95	0.12–7.08	0.93	
Ferrets	No	Ref	-		-	0.08
	Yes	0.37	0.23	0.11–1.27	0.11	
Feed storage prior to opening	Feed shed	Ref	-	-	-	0.81
	Shed and silo	0.43	0.45	0.06–3.33	0.42	
	Other shed	0.77	0.59	0.17–3.46	0.74	
	Other	1.09	0.62	0.36–3.35	0.88	
Feed storage once opened	Sealed feed bins	Ref	-		-	0.38
	Open feed bags	2.37	1.21	0.87–6.47	0.09	
	Unsealed feed bins	0.62	0.49	0.13–2.90	0.54	
	Silo	0.80	0.54	0.22–3.00	0.75	
	Other	1.19	0.95	0.25–5.65	0.83	

Animals on property						
Cats	No	Ref	-		-	0.96
	Yes	1.03	0.65	0.30–3.56	0.96	
Dogs	No	Ref	-		-	0.95
	Yes	1.06	1.11	0.14–8.26	0.95	
Contact with other animals						
Graze horses same time as cattle	No	Ref	-		-	0.53
	Yes	1.31	0.55	0.57–3.00	0.53	
Graze horses alternately with cattle	No	Ref	-		-	0.25
	Yes	0.86	0.38	0.36–2.06	0.74	
Graze horses same time as sheep	No	Ref	-		-	0.14
	Yes	1.87	0.79	0.82–4.27	0.14	
Graze horses alternately with sheep	No	Ref	-		-	0.39
	Yes	1.43	0.60	0.63–3.25	0.40	
Contact with deer over fence	No	Ref	-		-	0.82
	Yes	0.85	0.64	0.19–3.72	0.83	

Variables mice, traps on the property and goats were not modelled as they predicted success or failure perfectly. Correlated variables not shown.

Table S4. Results of univariable logistic regression investigating horse and property-level risk factors for seropositivity to Copenhageni (at cut-off ≥50), in a convenience sample of 499 racehorses and broodmares in New Zealand.

Risk Factor	Level	Odds Ratio	Robust SE	95% Confidence Interval	*p*-Value	LR *p*-Value
Age		1.12	0.04	1.06–1.19	<0.001	<0.001
Role	Racehorse	Ref	-	-	-	0.006
	Broodmare	0.35	0.15	0.15–0.80	0.001	
Vaccinated against other pathogens	No/unsure	-	-	-	-	0.16
	Yes	1.81	1.31	0.74–4.40	0.19	
Region	Manawatu	Ref	-	-	-	0.43
	Waikato	1.55	1.29	0.80–3.00	0.198	
	Auckland	1.05	0.59	0.35–3.17	0.93	
Natural water source on property	No	Ref	-	-	-	0.91
	Yes	1.05	0.48	0.43–2.58	0.92	
Flooding in the last 12 months	No	Ref	-	-	-	0.25
	Yes	0.7	0.22	0.37–1.30	0.26	
Evidence of wildlife on the property						
Rats	No	Ref	-	-	-	0.23
	Yes	1.74	0.85	0.67–4.54	0.26	
Mice	No	Ref	-	-	-	0.83
	Yes	0.79	0.85	0.10–6.46	0.83	
Possums	No	Ref	-	-	-	0.23
	Yes	1.20	0.47	0.56–2.58	0.63	
Hedgehogs	No	Ref	-	-	-	0.71
	Yes	1.12	0.35	0.61–2.07	0.71	
Ferrets	No	Ref	-	-	-	0.05
	Yes	0.47	0.19	0.20–1.07	0.07	
Feed storage prior to opening	Feed shed	Ref	-	-	-	0.52
	Shed and silo	0.43	0.32	0.10–1.87	0.26	
	Other shed	0.58	0.36	0.17–1.97	0.38	
	Other	0.98	0.43	0.41–2.31	0.96	
Feed storage once opened	Sealed feed bins	Ref	-	-	-	0.26
	Open feed bags	1.79	0.78	0.76–4.21	0.18	
	Unsealed feed bins	0.92	0.49	0.33–2.59	0.88	
	Silo	1.12	0.53	0.45-2.81	0.80	
	Other	2.74	1.34	1.05-7.12	0.04	
Animals on property						
Cats	No	Ref	-	-	-	0.39
	Yes	1.56	0.85	0.54–4.51	0.41	
Dogs	No	Ref	-	-	-	0.47
	Yes	0.61	0.39	0.17–2.15	0.44	
Goats	No	Ref	-	-	-	0.97
	Yes	0.97	0.61	0.29–3.32	0.97	

Contact with other animals						
Graze horses same time as cattle	No	Ref	-	-	-	0.76
	Yes	1.1	0.36	0.59–2.07	0.76	
Graze horses alternately with cattle	No	Ref	-	-	-	0.42
	Yes	0.77	0.25	0.40–1.46	0.41	
Graze horses same time as sheep	No	Ref	-	-	-	0.82
	Yes	1.08	0.22	0.56–2.06	0.82	
Graze horses alternately with sheep	No	Ref	-	-	-	0.26
	Yes	0.7	0.22	0.37–1.31	0.27	
Contact with deer over fence	No	Ref	-	-	-	0.07
	Yes	2.24	0.95	0.98–5.13	0.06	

Variables traps on the property and rabbits were not modelled as they predicted success or failure perfectly. Correlated variables not shown.

Table S5. Results of univariable logistic regression investigating horse and property-level risk factors for seropositivity to Hardjo(bovis), in a convenience sample of 499 racehorses and broodmares in New Zealand.

Risk Factor	Level	Odds Ratio	Robust SE	95% Confidence Interval	*p*-Value	LR *p*-Value
Age		1.08	0.04	0.99–1.16	0.05	0.06
Role	Racehorse	Ref	-	-	-	0.61
	Broodmare	0.81	0.35	0.35–1.87	0.62	
Vaccinated against other pathogens	No/unsure	Ref	-	-	-	0.96
	Yes	0.97	0.46	0.38–2.47	0.96	
Region	Manawatu	Ref	-	-	-	0.83
	Waikato	0.85	0.38	0.35–2.06	0.71	
	Auckland	0.68	0.51	0.15–3.00	0.61	
Natural water source on property	No	Ref	-	-	-	0.54
	Yes	0.72	0.37	0.27–1.97	0.53	
Flooding in the last 12 months	No	Ref	-	-	-	0.08
	Yes	0.49	0.2	0.22–1.11	0.09	
Evidence of Wildlife on the property						
Rats	No	Ref	-	-	-	0.39
	Yes	0.49	0.38	0.10–2.22	0.35	
Possums	No	Ref	-	-	-	0.23
	Yes	1.83	1.01	0.62–5.38	0.273	
Hedgehogs	No	Ref	-	-	-	0.49
	Yes	1.32	0.52	0.61–2.84	0.484	
Rabbits	No	Ref	-	-	-	0.39
	Yes	0.49	0.38	0.35	0.12-2.22	
Ferrets	No	Ref	-	-	-	0.3
	Yes	1.54	0.63	0.69–3.42	0.29	
Traps for rodents on property	No	Ref	-	-	-	0.39
	Yes	0.49	0.38	0.12–2.22	0.35	
Feed storage prior to opening	Feed shed	Ref	-	-	-	0.19
	Shed and silo	3.35	1.72	1.23–9.14	0.02	
	Other shed	1.35	0.88	0.38–4.86	0.64	
	Other	1.25	0.72	0.40–3.87	0.70	
Feed storage once opened	Sealed feed bins	Ref	-	-	-	0.54
	Open feed bags	-	-	-	-	
	Unsealed feed bins	1.63	0.84	0.59–4.50	0.35	
	Silo	1.89	0.90	0.74–4.79	0.18	
	Other	0.98	0.77	0.21–4.57	0.98	
Animals on property						
Cats	No	Ref	-	-	-	0.32
	Yes	1.97	1.47	0.46–8.50	0.36	
Goats	No	Ref	-	-	-	0.15

	Yes	2.45	1.4	0.80–7.51	0.12	
Contact with other animals						
Graze horses same time as cattle	No	Ref	-	-	-	0.11
	Yes	1.87	0.73	0.87–4.02	0.1	
Graze horses alternately with cattle	No	Ref	-	-	-	<0.001
	Yes	12.6	12.9	1.70–93.7	0.01	
Graze horses same time as sheep	No	Ref	-	-	-	0.66
	Yes	1.2	0.49	0.54–2.66	0.65	
Graze horses alternately with sheep	No	Ref	-	-	-	0.02
	Yes	2.65	1.09	1.17–5.97	0.02	
Contact with deer over fence	No	Ref	-	-	-	0.63
	Yes	0.71	0.53	0.16–3.09	0.65	

Variables dogs and mice were not modelled as they predicted success or failure perfectly. Correlated variables not shown.

Table S6. Results of univariable logistic regression investigating horse and property-level risk factors for seropositivity to Pomona, in a convenience sample of 499 racehorses and broodmares in New Zealand.

Risk Factor	Level	Odds Ratio	Robust SE	95% Confidence Interval	*p*-Value	LR *p*-Value
Age		1.19	0.05	1.10–1.29	<0.001	<0.001
Role	Racehorse	Ref	-	-	-	<0.001
	Broodmare	0.15	0.11	0.03–0.63	0.01	
Region	Manawatu	Ref	-	-	-	0.96
	Waikato	1.10	0.48	0.46–2.60	0.84	
	Auckland	1.17	0.76	0.33–4.15	0.81	
Natural water source on property	No	Ref	-	-	-	0.54
	Yes	0.72	0.37	0.27–1.97	0.52	
Flooding in the last 12 months	No	Ref	-	-	-	0.17
	Yes	0.58	0.24	0.18–0.26	1.29	
Evidence of wildlife on the property						
Rats	No	Ref	-	-	-	0.93
	Yes	0.96	0.49	0.35–2.59	0.93	
Possums	No	Ref	-	-	-	0.85
	Yes	1.09	0.52	0.43–2.76	0.85	
Hedgehogs	No	Ref	-	-	-	0.48
	Yes	1.32	0.52	0.61–2.84	0.48	
Ferrets	No	Ref	-	-	-	0.83
	Yes	1.08	0.47	0.47–2.52	0.85	
Feed storage prior to opening	Feed shed	Ref	-	-	-	0.66
	Shed and silo	-	-	-	-	
	Other shed	1.29	0.73	0.42–3.93	0.65	
	Other	0.65	0.41	0.19–2.23	0.49	
Feed storage once opened	Sealed feed bins	Ref	-	-	-	0.42
	Open feed bags	1.49	0.83	0.5–4.43	0.48	
	Unsealed feed bins	0.56	0.44	0.12–2.59	0.46	
	Silo	2.07	1.00	0.8–5.33	0.13	
	Other	1.08	0.85	0.23–5.05	0.93	
Animals on property						
Cats	No	Ref	-	-	-	0.43
	Yes	0.66	0.34	0.24–1.8	0.42	
Goats	No	Ref	-	-	-	0.04
	Yes	3.31	1.75	1.17–9.35	0.02	
Contact with other animals						
Graze horses same time as cattle	No	Ref	-	-	-	0.11
	Yes	1.87	0.73	0.87–4.02	0.11	
Graze horses alternately with cattle	No	Ref	-	-	-	0.12
	Yes	2.07	1.04	0.77–5.54	0.15	

Graze horses same time as sheep	No	Ref	-	-	-	0.66
	Yes	1.20	0.49	0.54–2.66	0.65	
Graze horses alternately with sheep	No	Ref	-	-	-	0.64
	Yes	1.20	0.47	0.56–2.57	0.64	
Contact with deer over fence	No	Ref	-	-	-	0.63
	Yes	0.71	0.53	0.16–3.09	0.65	

Variables mice, rabbits, dogs, vaccinated against other pathogens, and traps for rodents not modelled as they predicted success or failure perfectly. Correlated variables not shown.

Table S7: Results of univariable logistic regression investigating horse and property-level risk factors for seropositivity to Tarassovi, in a convenience sample of 499 racehorses and broodmares in New Zealand.

Risk Factor	Level	Odds Ratio	Robust SE	95% Confidence Interval	*p*-Value	LR *p*-Value
Age		1.15	0.04	1.07–1.24	<0.001	<0.001
Role	Racehorse	Ref	-		-	<0.005
	Broodmare	0.14	0.10	0.03–0.60	0.01	
Vaccinated against other pathogens	No/unsure	Ref	-	-	-	0.03
	Yes	3.78	2.80	0.88–16.1	0.07	
Region	Manawatu	Ref	-		-	0.84
	Waikato	1.48	0.62	0.65–3.36	0.745	
	Auckland	1.25	0.81	0.35–4.45	0.583	
Natural water source on property	No	Ref	-		-	1.00
	Yes	1.00	0.56	0.34–2.98	1.00	
Flooding in the last 12 months	No	Ref	-		-	0.72
	Yes	0.87	0.33	0.41–1.85	0.72	
Evidence of wildlife on the property						
Rats	No	Ref	-		-	0.60
	Yes	1.32	0.73	0.45–3.9	0.61	
Possums	No	Ref	-		-	0.45
	Yes	1.45	0.73	0.54–3.89	0.46	
Hedgehogs	No	Ref	-		-	0.20
	Yes	1.64	0.64	0.76–3.54	0.21	
Rabbits	No	Ref	-		-	0.11
	Yes	0.31	0.20	0.08–1.12	0.07	
Ferrets	No	Ref	-		-	0.95
	Yes	1.03	0.44	0.44–2.38	0.95	
Feed storage prior to opening	Feed shed	Ref	-		-	0.69
	Shed and silo	0.36	0.38	0.05–2.78	0.33	
	Other shed	1.00	0.64	0.29–3.49	1.00	
	Other	1.17	0.60	0.42–3.22	0.76	
Feed storage once opened	Sealed feed bins	Ref	-		-	0.86
	Open feed bags	0.67	0.44	0.19–2.40	0.54	
	Unsealed feed bins	0.66	0.43	0.18–2.37	0.53	
	Silo	1.17	0.59	0.44–3.16	0.75	
	Other	1.29	0.85	0.35–4.70	0.70	
Animals on property						
Cats	No	Ref	-		-	0.67
	Yes	1.29	0.81	0.38–4.40	0.68	
Dogs	No	Ref	-		-	0.53
	Yes	0.60	0.46	0.13–2.70	0.51	
Goats	No	Ref	-		-	0.99
	Yes	1.01	0.77	0.23–4.46	0.99	
Deer	No	Ref	-		-	0.89

	Yes	1.09	0.69	0.32–3.74	0.89	
Contact with other animals						
Graze horses same time as cattle	No	Ref	-		-	0.89
	Yes	0.95	0.38	0.43–2.08	0.89	
Graze horses alternately with cattle	No	Ref	-		-	0.03
	Yes	2.84	1.56	0.97–8.32	0.06	
Graze horses same time as sheep	No	Ref	-		-	0.35
	Yes	0.67	0.30	0.28–1.59	0.36	
Graze horses alternately with sheep	No	Ref	-		-	0.50
	Yes	1.29	0.50	0.61–2.74	0.50	
Contact with deer over fence	No	Ref	-		-	0.89
	Yes	1.09	0.69	0.32–3.74	0.89	

Variables mice and traps for rodents not modelled as they predicted success or failure perfectly. Correlated variables not shown.
